# Unique mechanism of target recognition by PfoI restriction endonuclease of the CCGG-family

**DOI:** 10.1093/nar/gky1137

**Published:** 2018-11-16

**Authors:** Giedre Tamulaitiene, Elena Manakova, Virginija Jovaisaite, Gintautas Tamulaitis, Saulius Grazulis, Matthias Bochtler, Virginijus Siksnys

**Affiliations:** 1 Institute of Biotechnology, Vilnius University, Sauletekio al. 7, LT-10257 Vilnius, Lithuania; 2 Laboratory of Structural Biology, International Institute of Molecular and Cell Biology, Trojdena 4, 02–109 Warsaw, Poland; 3 Dept. of Bioinformatics, Institute of Biochemistry and Biophysics, Polish Academy of Sciences, Pawinskiego 5a, 02–106 Warsaw, Poland

## Abstract

Restriction endonucleases (REs) of the CCGG-family recognize a set of 4–8 bp target sequences that share a common CCGG or CCNGG core and possess PD…D/ExK nuclease fold. REs that interact with 5 bp sequence 5′-CCNGG flip the central N nucleotides and ‘compress’ the bound DNA to stack the inner base pairs to mimic the CCGG sequence. PfoI belongs to the CCGG-family and cleaves the 7 bp sequence 5′-T|CCNGGA ("|" designates cleavage position). We present here crystal structures of PfoI in free and DNA-bound forms that show unique active site arrangement and mechanism of sequence recognition. Structures and mutagenesis indicate that PfoI features a permuted E…ExD…K active site that differs from the consensus motif characteristic to other family members. Although PfoI also flips the central N nucleotides of the target sequence it does not ‘compress’ the bound DNA. Instead, PfoI induces a drastic change in DNA backbone conformation that shortens the distance between scissile phosphates to match that in the unperturbed CCGG sequence. Our data demonstrate the diversity and versatility of structural mechanisms employed by restriction enzymes for recognition of related DNA sequences.

## INTRODUCTION

Type II restriction endonucleases (REs) recognize short nucleotide sequences typically 4–8 bp in length and cut both DNA strands within or close to their target sites generating a double strand break. Restriction enzymes exhibit extreme specificity: change of a single base within a target site reduces k_cat_/K_M_ by 10^6^-fold or more (1–3). Type II REs recognize more than 450 different target sequences and show a high level of protein sequence diversity and a variety of strategies of DNA recognition and cleavage (4,5).

**Figure 1.**
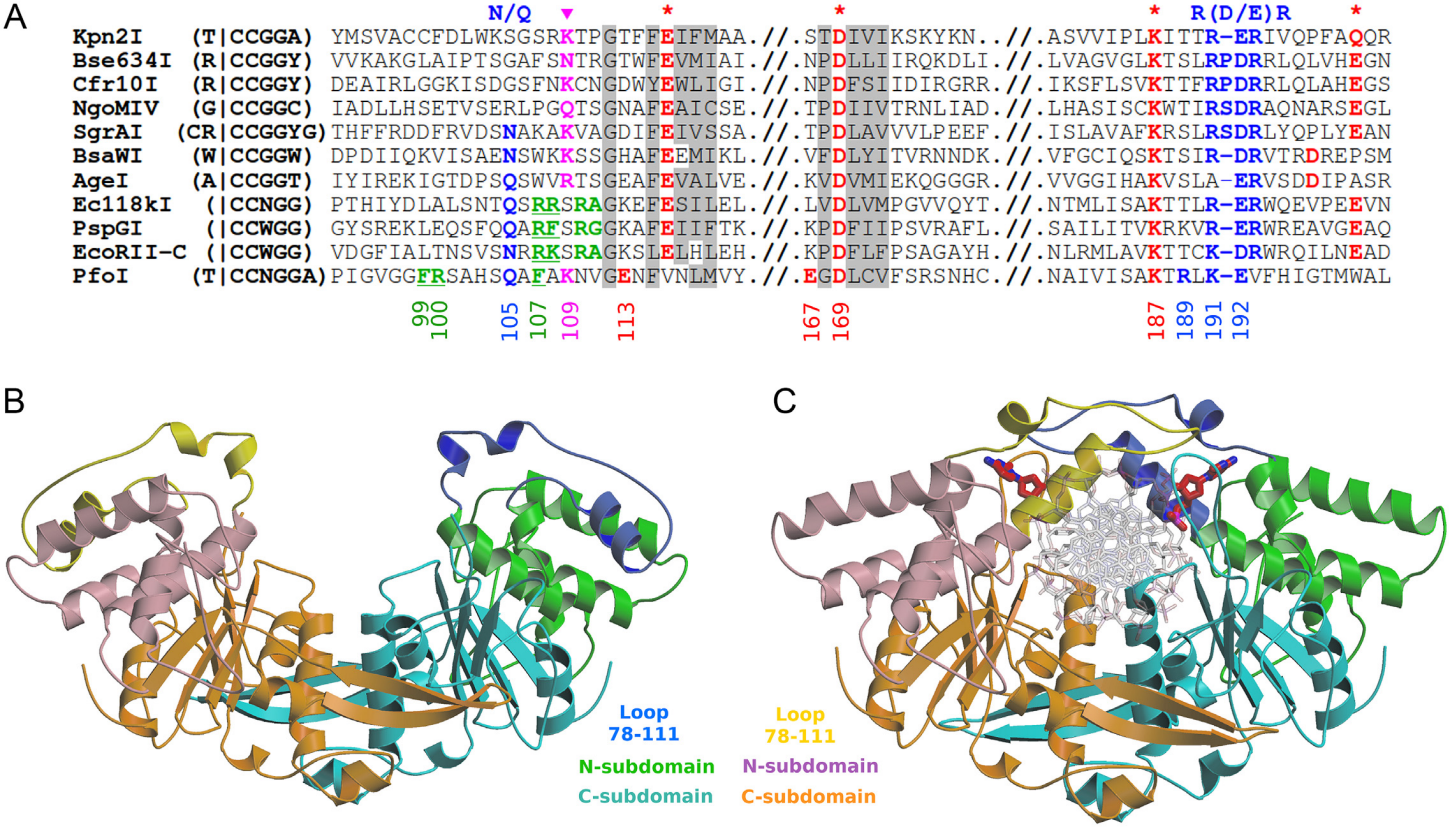
The crystal structures of apo- and DNA-bound PfoI. (**A**) The partial primary protein sequence alignment of the conserved motifs of CCGG-RE family members. Note, that alignment of PfoI was adjusted after structure determination, before that, only D169 and K187 of active site motif and K191 and E192 from CCGG recognition motif could be aligned. Conserved residues in the family are shadowed in gray. Active site residues (highlighted red) are marked by stars. Residues that interact with the outer base pair of the recognition sequence (magenta) are marked by magenta triangles. The conserved CCGG recognition motif R-(D/E)R is shown in blue. Residues that form the pocket for a flipped base are highlighted green and residues displacing the flipped central base are underlined. The numbering of important PfoI residues is shown below the alignment. (**B**) The crystallographic dimer of apo-PfoI. Subunits are colored orange-pink-yellow and cyan-green-blue. N-subdomains (residues 1–78 and 111–131) are pink and light green, C-subdomains (residues 132–307) are orange and cyan. Residues 78–111 that are swapped between the protomers in the PfoI–DNA complex are shown in yellow and blue. (**C**) The dimer of PfoI–DNA complex, coloring is the same as in (B). DNA is shown in gray, the flipped bases are highlighted in red.

In order to study target recognition by restriction enzymes, we have focused on a family of related proteins that recognize target sites containing the conserved 5′-CCGG/5′-CCNGG sequence and cut it before the first C. The CCGG-family now contains 11 structurally and biochemically characterized REs recognizing 4–8 bp sequences bearing CCGG in different nucleotide sequence contexts: 5′-R|CCGGY ("|" designates cleavage position) (Cfr10I (6), Bse634I (7)), 5′-G|CCGGC (NgoMIV (8)), 5′-CR|CCGGYG (SgrAI (9)), 5′-W|CCGGW (BsaWI (10)), 5′-A|CCGGT (AgeI (11)), 5′-T|CCGGA (Kpn2I), 5′-|CCWGG (PspGI (12)), EcoRII (13)) and 5′-|CCNGG (Ecl18kI (14)). Mutational studies and crystal structures have revealed that REs of the CCGG-family share a permuted active site variant PD…K…D/E that differs from the canonical PD…D/ExK catalytic motif (Figure 1A) (6,15–17). Structural comparisons show that these REases also share a conserved mechanism of the CCGG tetranucleotide recognition. The N-terminal end of a conserved α-helix projects into DNA major groove and conserved amino acid residues R-(D/E)R make base specific contacts to the donor-acceptor atoms on base edges (Figure 1A). Specifically, the side chain oxygen atoms of the acidic residue (D/E) accept one hydrogen bond each from the two neighboring cytosines, while two arginines donate bidentate H-bonds to the outer and inner G bases of one recognition half-site, respectively (8,14,18). This recognition pattern is also conserved for REs that interact with an interrupted CCGG target. Such REs flip the central base pair out of the duplex and compress DNA to make the 5 bp duplex mimic a 4 bp duplex. The mimicry provides a rationale for the conservation of the structural and molecular mechanisms of the CCGG-tetranucleotide recognition within the RE family (12–14). Despite the commonalities, CCGG-REs are strikingly diverse in their oligomeric structures and DNA cleavage mechanisms (reviewed in (11)).

PfoI, identified in *Pseudomonas fluorescens* biovar 126, recognizes the 7 bp sequence 5′-T|CCNGGA (19). PfoI shows no significant protein sequence similarity with CCGG-REs, except of few residues from the catalytic and CCGG recognition motifs that could be manually aligned (Figure 1A). Fluorescence lifetime experiments demonstrate that PfoI flips 2-aminopurine (2AP) embedded in the 5′-TCC(2AP)GGA target like other enzymes that recognize an interrupted CCNGG sequence (20,21). The related target sequence, cleavage pattern, the distant amino acid sequence similarity and nucleotide flipping all suggest that PfoI belongs to the CCGG REs.

Here, we present crystal structures of PfoI in DNA-bound and apo-form. We show that, in comparison to other family members, the active site and CCGG recognition motifs of PfoI are permutated, demonstrating the structural plasticity in the target sequence recognition and cleavage. We further show that PfoI shares the mechanism of the outer T:A base pair recognition with AgeI, which recognizes the 5′-A|CCGGT sequence. We provide a crystallographic demonstration that PfoI flips out central N nucleotide from its target, but does not compress DNA in the same way as other REs of the same family. PfoI induces a drastic change in DNA backbone conformation that changes the distance between scissile phosphates to match the distance in the unperturbed CCGG sequence.

## MATERIALS AND METHODS

### Oligonucleotides

All oligonucleotides (Supplementary Table S1) were synthesized and HPLC purified by Metabion (Martinsried, Germany). Oligoduplexes were assembled by slow annealing from 90°C to the room temperature in 50 mM Tris–HCl, pH 8.0. For DNA binding studies oligoduplexes were labeled by T4 polynucleotide kinase (Thermofisher, Vilnius, Lithuania) at 5′-end with [γ-^33^P]ATP (Hartmann Analytic, Braunschweig, Germany).

### Protein expression and purification

PfoI was expressed in the *E. coli* strains BL21(DE3) or DH10B carrying the methyltransferase gene in the plasmid pACYC184-M.Ecl18kI (Cm^r^) and pBAD24-R.PfoI (Ap^r^). The protein expression was induced by 0.2% arabinose and the culture in LB medium was grown at 37°C. The protein was expressed for 3–4 h. All mutant variants of PfoI were created using the same expression system. Mutant variants were constructed using modified QuickChange method (22). Introduced mutations were checked by sequencing of the protein coding region of the corresponding plasmid. Se-methionine labeled PfoI mutant variant K187A was expressed in mineral M9 medium using glucose as a carbon source. The overnight culture was diluted 1:10 into fresh medium and was grown till the mid-log phase during 5–6 h at 37°C. Amino acids that suppress methionine synthesis pathway were added to the cell culture 15–20 min before induction by 0.2% arabinose as described in (23). The protein expression was allowed to proceed overnight.

Wild-type (WT) PfoI as well as mutant proteins were purified according to the protocol described in (19), with modifications. We carried out sequential chromatographies on HiTrap Heparin and MonoQ 5/50 columns (GE Healthcare) in the buffer containing 10 mM potassium phosphate pH 7.4, 1 mM Ethylenediaminetetraacetic acid ( EDTA), 100 mM NaCl and 5 mM β-mercaptoethanol. Proteins were eluted by gradient of NaCl. Purified PfoI and mutants were dialyzed against the storage buffer containing 20 mM Tris–HCl pH 7.5, 300 mM KCl, 1 mM dithiothreitol (DTT), 0.1 mM EDTA and 50% (v/v) glycerol and stored at −20°C.

Concentrations of the protein monomers were determined by measuring absorption at 280 nm using the extinction coefficient 35143 M^−1^cm^−1^ for PfoI, as calculated by the ProtParam tool (http://web.expasy.org/protparam/, (24).

### Protein crystallization

All the crystallization trials were carried out by sitting drop vapor diffusion method at 19°C. The crystals of apo-PfoI were grown only in the presence of DNA. Two different crystal forms were obtained. PfoI-SP12 (Supplementary Table S1) complex crystals in the P4_3_2_1_2 space group (2.6 Å resolution) were produced by mixing of the PfoI-SP12 complex (6.8 mg/ml of PfoI) with the reservoir solution containing 0.1 M Na-Hepes (4-(2-hydroxyethyl)-1-piperazineethanesulfonic acid) (pH 7.0-7.5), 0.2 M magnesium formate and 18-20% (w/v) PEG3350. Crystals of the suitable size were grown using microseeding with the help of Oryx8 robot (Douglas Instruments). P2_1_2_1_2_1_ crystals (3.0 Å resolution) were obtained with oligoduplex SP11 (Supplementary Table S1) by mixing of PfoI-SP11 complex with the crystallization buffer containing 0.2 M magnesium nitrate and 20% (w/v) PEG3350, pH 5.9 (Hampton Research, PEG-Ion screen #16). Only the crystal structure of higher resolution was solved.

The Se-methionine labeled PfoI K187A mutant was dialyzed into crystallization buffer (300 mM KCl, 20 mM Tris–HCl pH 7.5 and 1 mM DTT) and mixed with the oligoduplex SP14 in a dimer to duplex ratio 1:1.2 (Supplementary Table S1). The final concentration of the protein was 5.3 mg/ml. PfoI-SP14 complex (0.4 μl) was mixed with 0.3 μl of reservoir solution containing 20% (w/v) of PEG8000, 100 mM Tris–HCl pH 8.5, 200 mM LiCl and 10% (v/v) of glycerol. Crystallization plates were prepared with the help of Oryx8 crystallization robot (Douglas Instruments) and 0.1 μl of the microcrystal seed solution was added into each drop using the ‘microseeding approach’ (25,26). Crystal growth took approximately three months.

### Data collection and structure determination

The apo-PfoI P4_3_2_1_2 dataset was collected to the nominal resolution 2.6 Å on the Rigaku RU H3R X-ray generator (Rigaku, Japan) equipped with RAXIS IV++ detector. Before data collection the crystal was flash-cryocooled after soaking in the cryo-protecting solution, that was prepared by adding 10% (v/v) ethylene glycol to the reservoir solution.

The native data were integrated with MOSFLM (27)), and further processed by SCALA (28) and TRUNCATE (29). Initial phases were obtained using MOLREP (30). COOT (31), REFMAC (32,33) and PHENIX (34) were used for model improvement. Molecular representations were made with MOLSCRIPT and RASTER3D (35,36). Dimer interfaces were analyzed by PISA (37). Secondary structures were analyzed by PDBsum suite (38). Conformational parameters of DNA bound to PfoI and other CCGG-REs were assessed by w3DNA server (http://w3dna.rutgers.edu/, (39)).

The MAD dataset of Se-methionine labeled K187A mutant of PfoI in complex with oligoduplex SP14 was collected at the macromolecular crystallography beamline I911-3 at the MAX IV laboratory of Lund University (Sweden). Before data collection crystal was quickly passed through mineral oil (Sigma-Aldrich) removing the rest of the crystallization solution and flash-cryocooled in the 100 K nitrogen stream. The data collection, refinement statistics and PDB access codes are presented in Table 1.

**Table 1.** PfoI data collection and refinement statistics

**Data collection statistics**	**apo-PfoI**	**PfoI-DNA**
Dataset	PfoI	SeMet-PfoI –DNA complex
DNA oligoduplex	SP12 (no DNA in the structure)	SP14
Space group	P 4_3_ 2_1_ 2	P 2_1_ 2_1_ 2_1_
Cell constants	*a* = *b* = 48.0 Å, *c* = 266.6 Å, *α* = *β* = *γ* = 90°	*a* = 56.28 Å, *b* = 91.66 Å, *c* = 152.74 Å, *α* = *β* = *γ* = 90°
Wavelength, Å	1.5418	0.96112, 0.98010
X-ray source	Rigaku RU H3R	I911–3 at the MAX IV laboratory of Lund University
Total reflections: overall (outer shell)	54 361 (9146)	487 954 (28 642)
Unique reflections: overall (outer shell)	10 283 (1470)	36 181 (4683)
Resolution range, Å	2.6–45.22	2.28–47.96
Completeness: overall (outer shell)	98.6 (100)	98.5 (89.8)
Multiplicity: overall (outer shell)	5.3 (6.2)	13.5 (6.1)
Anomalous multiplicity: overall (outer shell)		7.2 (3.2)
I/σ: overall (outer shell)	15.5 (3.4)	11.6 (1.8)
R_merge_: overall (outer shell), %	11.2 (44.0)	19.8 (97.4)
B-factor from Wilson, Å^2^	38.3	27.3
**Refinement statistics**		
Resolution range, Å	2.6–45.22	2.28–47.96
Reflections: work (non-anomalous)/test	17 795 / 1710	68 055 / 6650
Atom number: protein/DNA /solvent	2406 /- / 10	4919 / 564 / 266
R_cryst_/R_free_, %	22.3 / 27.4	20.9 / 24.8
RMSD: bond lengths, Å / bond angles, (°)	0.002 / 0.574	0.009 / 1.085
Ramachandran: favoured/allowed/outliers, %	95 / 5 / 0	96 / 4 / 0.2
Average B-factors: all atoms / main chain / side chain / DNA / solvent, Å^2^	41.0 / 41.4 / 42.4 / - / 30.2	33.0 / 29.6 / 31.9 / 24.4 / 29.5
**PDB ID**	**6EK1**	**6EKO**

The dataset was processed by XDS (40). The structure was solved using 2W-MAD protocol of Auto-Rickshaw, the EMBL-Hamburg automated crystal structure determination platform (41). The input diffraction data were prepared and converted for use in Auto-Rickshaw using programs of the CCP4 suite (29). FA structure factors values were calculated using the program SHELXC (42). Heavy atom positions were found using the program SHELXD (43). The correct hand for the substructure was determined using the program ABS (44) and initial phases were calculated by SHELXE (45). The initial phases were improved using the density modification program DM (46). The initial model was built by the program ARP/wARP (47,48). DNA density was clearly visible in the initial model and nucleotides were built into the model manually in COOT. The model was improved by several cycles of refinement in REFMAC and PHENIX and manual inspection in COOT.

### DNA binding experiments

Specific and unspecific DNA binding by WT PfoI and mutants was studied using gel mobility shift assay. Radio labeled specific and unspecific oligoduplexes of the same length SP25 and NSP25 (Supplementary Table S1) at 0.2 nM were mixed with increasing concentrations of protein (0.05–1000 nM of dimer) in 20 μl of 40 mM Tris-acetate pH 8.3, 0.1 mg/ml of bovine serum albumin, 5 mM calcium acetate and 10% (v/v) glycerol. After 15 min of incubation at the room temperature free DNA and protein-DNA complexes were resolved by electrophoresis in 8% polyacrylamide gel (29:1 acrylamide to N,N'–methylenebisacrylamide ratio) at 6 V/cm for 2–2.5 h. Gels were visualized by Cyclone Phosphor-Imager and quantified by OptiQuant software (Packard Instrument, USA).

### DNA cleavage assays

According to the definition (ThermoFisher catalog), 1 unit of specific activity of the RE is the amount of the protein that digested completely 1 μg of λ DNA in 1 h in 50 μl of reaction volume. The Reaction mix contained 33 mM of Tris-acetate (pH 7.9 at 37°C), 33 mM potassium acetate, 10 mM magnesium acetate, 0.1 mg/ml bovine serum albumin, 0.5 μg *λ*^dam- dcm−^ DNA in 50 μl and sequential dilutions of the corresponding mutants (from undiluted to 1:1000). Cleavage reactions were carried out for 1 h at 37°C. Reactions were stopped by adding of 25 μl of the STOP solution (75 mM EDTA, sodium dodecylsulfate 0.3% (w/v), glycerol to 50% (v/v) and Bromophenol Blue 0.2% (w/v)) and incubated at 65°C for 10 min. Completeness of DNA digestion was analyzed in 0.8% agarose gels in running buffer that contained 0.1 M sodium borate, 0.2 mM EDTA, pH 8.0 and 0.5 μg/ml of ethidium bromide).

Cleavage of pUC18 plasmid (2.5 nM) containing a single PfoI site was performed at +15°C in the Reaction mix. For E<S conditions reaction contained 0.25 nM PfoI. For stimulation experiments WT PfoI was added to the concentration 125 nM in terms of dimer, and oligoduplex SP23 containing PfoI target (Supplementary Table S1) was added in-trans to the concentration 200 and 2000 nM. Reactions were initiated by adding Mg^2+^ ions. Aliquots were removed at defined time intervals (6–320 s). The reaction was quenched by adding 1/3 volume of STOP solution and products of cleavage were analyzed in 0.8% agarose gel. The amount of supercoiled (SC), open-circular (OC), and linear DNA forms (FLL) was evaluated by densitometric analysis of ethidium bromide-stained gels. Data analysis used the KYPLOT 2.0 software. An exponential function was fitted to the supercoiled plasmid depletion curves and apparent first-order reaction rate constants (*k_obs_*) were determined. The preparation of supercoiled pUC18 DNA used in this study contained 15% of the randomly nicked OC form. Assuming that randomly nicked DNA is equivalent to the intact substrate, we corrected the experimentally determined amounts of supercoiled (SC) and nicked (OC) DNA at each time point using the following equations: [SC]_corrected_ = [SC]_experimental_/0.85, [OC]_corrected_ = 100%-[SC]_corrected_-[FLL]_experimental_ (49).

### Estimation of the oligomeric state by gel filtration and DLS

Oligomeric state of apo-PfoI and PfoI–DNA complex was analyzed by analytical gel filtration on Superdex75 10/300 column (GE Healthcare). Two variants of buffer containing 10 mM Tris–HCl pH 7.5 and 5 mM calcium chloride were used for gel filtration. The ‘low salt’ buffer contained 150 mM NaCl and the ‘high salt’ buffer was supplemented with 300 mM KCl. In the ‘low salt’ buffer apo-PfoI showed undesirable interaction with the column, therefore the experiment was repeated in the ‘high salt’ buffer. The 100 μl sample containing 13.8 μM of PfoI dimer with or without DNA oligoduplex SP14-GF (Supplementary Table S1) in a ratio 1:1.2 was applied on the column. Protein elution was monitored by absorbance at 280 and 260 nm. Molecular weights were estimated according to the retardation times of protein standards (GE Healthcare) (Supplementary Table S2).

Dynamic light scattering (DLS) was performed at the various PfoI-SP14 complex concentrations using the Malvern Zetaseizer μV (Malvern Instruments, UK) and MW estimated from the apparent particle diameter by the Malvern software from the distribution analysis (Supplementary Table S3).

## RESULTS

### Crystal structures of PfoI

We solved the crystal structure of apo-PfoI at 2.6 Å resolution. Interestingly, the apo-PfoI crystals grow only in the presence of the specific oligoduplex that was not present in the crystal. In the crystal only one protein chain is present in the asymmetric unit; the dimer (Figure 1B) is obtained by applying crystallographic symmetry. Residues 239–249 are unresolved in the structure.

After numerous trials to crystallize PfoI with oligoduplexes of various lengths containing 3′- or 5′-T overhangs or blunt ends, diffracting crystals of the PfoI–DNA complex were obtained using 14 bp oligoduplex SP14 containing the C:C mismatch at the central base pair and 3′-T overhang (Supplementary Table S1). Crystals were grown in the presence of Ca^2+^ ions that do not support catalysis. They contain both polypeptide chains of the PfoI dimer, and non-cleaved, specifically bound double-stranded DNA in the asymmetric unit (Figure 1C). The data collection and refinement statistics of apo- and DNA-bound PfoI are presented in Table 1.

### Monomer structure

The overall fold of PfoI monomer represents a typical PD…D/ExK nuclease fold with the structural core made of central four-stranded β-sheet surrounded by α-helices (50–52). The PfoI monomer is composed of an N-terminal α-helical subdomain (residues 1-131, colored pink-yellow and blue-green in Figure 1B and C) and the C-terminal subdomain (residues 132–312) that bears PD…D/ExK core (colored orange and cyan in Figure 1B and C). According to the assignment of secondary structure elements by PDBsum (Supplementary Figure S1), the structural core of PfoI consists of β3, β6, β7, β9, β10 and α-helices α8, α9 and α12 and coincides with the conserved core of NgoMIV restriction enzyme (Supplementary Figure S2). Structurally, PfoI is most similar to AgeI (5DWB with DALI (53) Z-score 13.8) and PspGI (3BM3 with DALI Z-score 13.0).

### Dimer structure

In the apo-PfoI crystal structure two monomers related by the crystallographic symmetry (the symmetry operator: y-1,x+1,-z) form a dimer (Figure 1B). The dimer has the U-shape typical for other related REs. The dimer interface as calculated by PISA extends over ∼1890 Å^2^. Three different regions of the C-subdomain contribute to the dimer interface: a β-hairpin (residues 151–167), α-helix (residues 192–218) of the conserved RE core (Supplementary Figure S1A), and a region including residues 255–266, 278–282 and 288–293 that interacts with the β-hairpin of the other subunit. In total, the interface contains 26 hydrogen bonds and two salt bridges. In the PfoI–DNA complex (Figure 1C) the dimer interface is increased to ∼2230 Å^2^. An additional interface is formed by the N-subdomains (residues 80–82, 94–100 and 103–111) that completely encircle bound DNA. PfoI also forms dimers in solution, as demonstrated by analytical gel filtration and DLS measurements (Supplementary Tables S2 and S3 and Supplementary Figure S3).

### PfoI conformational changes induced by DNA binding

In the complex with cognate DNA, the N-subdomain of PfoI undergoes significant structural change due to the intersubunit swapping of loops (residues 78–111) that in the apo-form do not contribute to the dimer interface (Figure 1B and C, Supplementary Figure S4). Such loop swapping fully encloses DNA in the PfoI dimer. Additional conformational change occurs in the region 99–127, where two α-helices α6′ (residues 99–104) and α6 (residues 110–127) rearrange into a continuous α6 helix (residues 101–127) in the PfoI–DNA complex. Amino acid residues located in the region corresponding to the loop between α6′ and α6 in apo-PfoI, are involved in catalysis and recognition of DNA substrate (Supplementary Figure S1).

Residues 239–249 are disordered in the apo-PfoI structure. In the complex with DNA these amino acids form a loop which is involved in DNA recognition (DNA recognition loop). With the exception of this loop, the Cα atoms of the C-subdomains in the DNA-free and –bound forms can be superimposed with an root mean square deviation (RMSD) of ∼0.9 Å. In contrast, the N-subdomains change much more significantly. This is particularly evident for the swapped region (residues 78–111), but even the Cα atoms in the rest part of the N-subdomain (residues 1–78 and 113–133) superimpose only with an RMSD of ∼4.2 Å. The conformational flexibility of the N-terminal subdomains has been reported for several other CCGG-REs (7).

The dimer interface of apo-PfoI differs from that in the DNA-bound form. An additional interface is formed due to the swapping of the loops (residues 78–111) in the N-subdomains. Moreover, the dimer interface between the C-subdomains also changes slightly according to PISA analysis due to a slightly different orientation of the C-subdomains in the dimer (Supplementary Figure S4A).

### Active center organization

**Figure 2.**
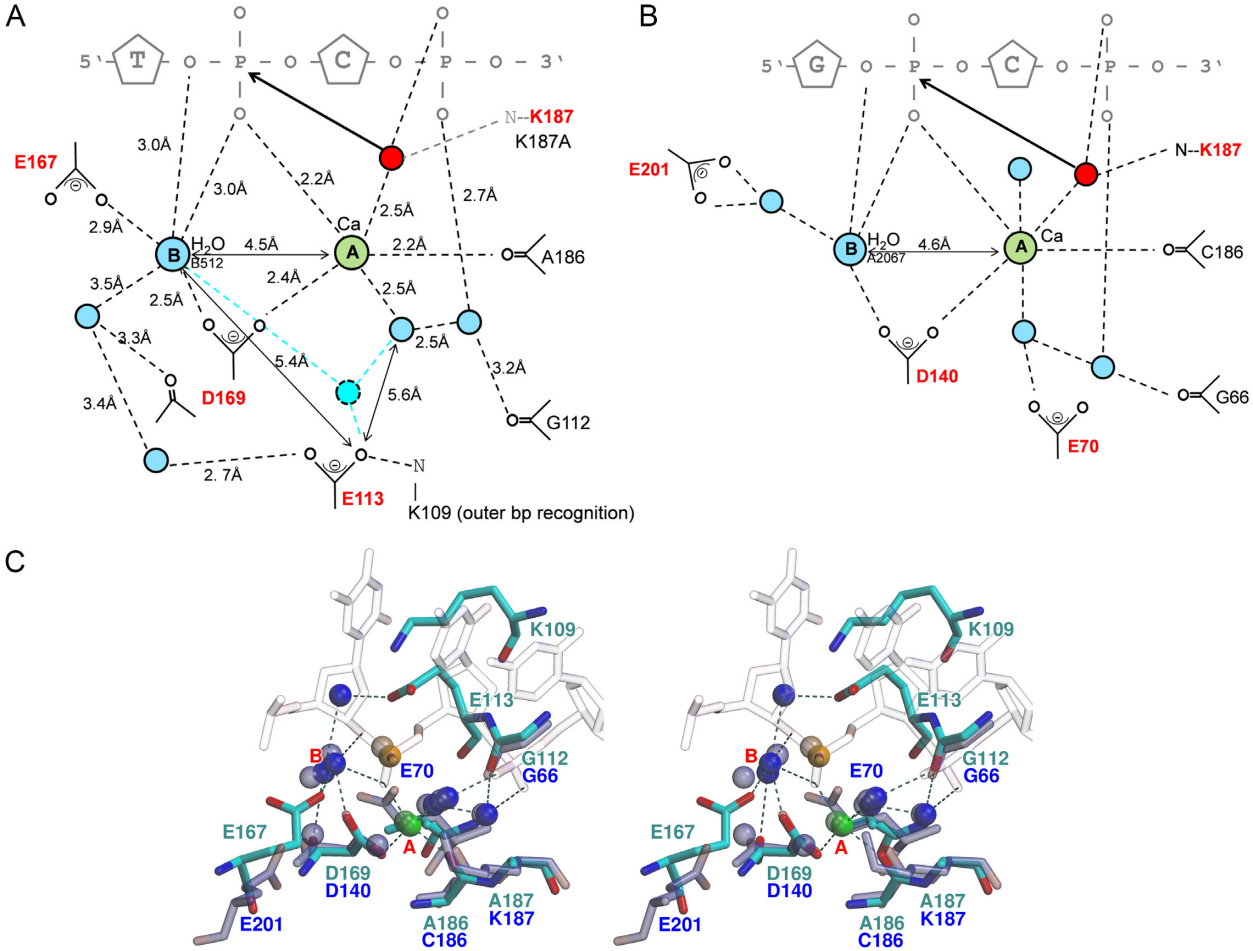
Proposed active center. (**A** and **B**) Schematic views of the active centers of PfoI (A, protein chain B of PfoI–DNA complex) and NgoMIV (B, PDB ID 4ABT). Positions of metal ions A and B are shown, water molecules are colored blue. The solvent molecule posed for in-line attack is shown red. In the co-crystal structures, the site of metal A is occupied by a Ca^2+^ ion (green), and the site for metal B is occupied by a water molecule (blue). When two Mg^2+^ ions are bound in the active site, the Mg^2+^ ion in site B may complete its coordination sphere with an additional water molecule (cyan), which may be co-anchored by E113. (**C**) Stereoview of overlaid catalytic residues of PfoI (cyan) and NgoMIV (PDB ID 4ABT, transparent blue). The scissile phosphate is shown as an orange sphere, Ca^2+^ ions bound in the active center is shown as a green sphere, water molecules are blue spheres.

The protein sequence of PfoI shows no significant similarities to other REs of the CCGG-family or any other protein except one homolog in a recently sequenced *Bacillus sp*. genome (WP_056521878, uncharacterized protein from *Bacillus sp*.), which shares 54% identity and 72% similarity with PfoI. Manual alignment of the PfoI sequence identified only conserved active site aspartate (D169) and lysine (K187) that match the conserved residues in other family members (Figure 1A). Other PfoI active site residues could not be predicted from the sequence alignment. The PfoI structure comparison with NgoMIV and other family members identified the residues E113, D169, K187 and E167 as potential PfoI catalytic/metal coordinating residues (Figure 2). Structurally, the E113 glutamate residue of the PfoI catalytic E^113^…E^167^xD^169^…K^187^ motif is located on a conserved N-terminal α-helix, which approaches DNA substrate from the minor grove, similarly to NgoMIV of the CCGG-family. However, E113 of PfoI is shifted one helical turn upstream from E70 of NgoMIV that is conserved in other CCGG-family members and is located at ∼7.3 Å from Ca^2+^ ion (Figures 1A and 2A). The position of glutamate E167 of the PfoI catalytic motif is also atypical (Figures 1A, 2A and 2C). E167 of PfoI is located close to the active site aspartate D169, on a loop that links a β-strand unique to PfoI (residues 158–166) to a β-strand that is part of the core (residues 169–173) (Supplementary Figure S1A and S1B).

The PfoI–DNA complex was crystallized in the presence of Ca^2+^ ions, which do not support catalysis. Altogether four Ca^2+^ ions are found in the structure, two in the active sites of the protomers, and two additional ones on the surface of one of the protomers. In each of the protomers, the active site Ca^2+^ ion is octahedrally coordinated, by the scissile phosphate, three water molecules, the main chain carbonyl of the A186 residue (preceding the conserved catalytic lysine) and carboxyl of D169 (coincides with D140 of NgoMIV). One of the water molecules that coordinate the Ca^2+^ (red in Figure 2A) is in a position just slightly too distant for in-line attack on the scissile phosphate, showing that the Ca^2+^ occupies the canonical metal A-binding site (54–56). This water molecule should also be coordinated by active site lysine K187, which is absent in the structure due to K187A mutation.

To confirm the functional importance of putative PfoI active site residues, we replaced these residues separately by alanines and analyzed DNA binding and cleavage properties of the protein variants (see, Table 2 and Supplementary Figure S5A and S5B). Active site mutants E167A, D169A and K187A show nearly no cleavage activity, whereas E113A shows ∼50-fold decrease in the cleavage activity compared to WT PfoI (Table 2). DNA binding by E167A and K187A mutants is similar to that of WT protein (Supplementary Figure S5A and S5B). DNA binding of E113A and D169A variants is significantly impaired. The E113A and D169A variants do not form a complex with the non-specific oligoduplex that lacks PfoI target.

**Table 2.** Specific activity and DNA binding of PfoI mutants

Mutation	Specific activity*, % WT	Specific DNA binding** Kd, nM	Mobility of the complex with specific DNA on gel	Unspecific DNA binding Kd, nM	Mobility of the complex with unspecific DNA on gel
WT	100	1	SP***	100	NSP****
**Active center mutants**					
E113A	2	100	SP	>1000	no complex
E167A	0	10	SP	1–10	NSP
D169A	0	100	SP	100	no complex
K187A	0	1	SP	100	NSP
**Central base pair pocket mutants**					
F99A	no complete cleavage	100	NSP	100	NSP
R100A	0	10	NSP	10	NSP
F99A-R100A	0	100	NSP	100	NSP

* Specific activity was estimated as a number of RE units per mg of protein (see ‘Materials and Methods’ section). Specific activity of WT was set to 100%.‘0’ means that no DNA cleavage products were observed after incubation of any amount of the protein used; ‘no complete cleavage’, only partial cleavage of DNA was observed even with maximal amount of the protein. Experiments were repeated twice.

** Kd values of specific and unspecific DNA binding were estimated as a concentration of PfoI variants at which half of the oligoduplex (SP25 and NSP25, Supplementary Table S1) is bound by protein as described in ‘Materials and Methods’ section. Experiments were repeated twice.

*** SP designated specific complex with DNA that has a higher mobility on native PAGE.

**** NSP is, correspondingly, non-specific complex with DNA that has lower mobility on native PAGE.

### Recognition of the CCGG-tetranucleotide

**Figure 3.**
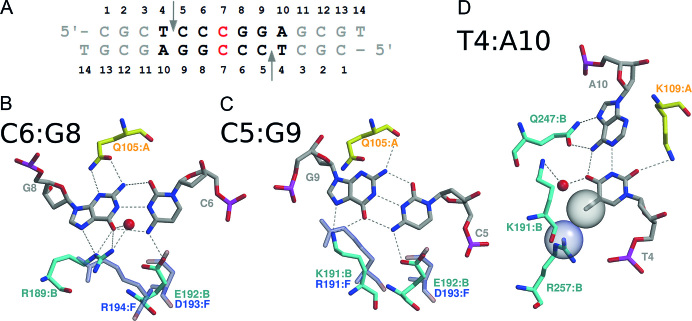
Specific DNA recognition by PfoI. (**A**) Sequence and base numbering of the oligoduplex in the PfoI–DNA complex. (**B** and **C**) CCGG-tetranucleotide recognition by PfoI. (**D**) Specific interaction with the outer base pair. Residues are superimposed on the corresponding residues of NgoMIV (PDB ID 4ABT, transparent blue). PfoI side chains that interact with DNA from the major groove are shown in cyan, and those from the minor groove - yellow, following monomer coloring as in (Figure 1).

One PfoI subunit interacts with one half-site of the CCGG tetranucleotide. The N-terminal end of the PfoI helix α8 projects into DNA major groove and amino acid residues R189, K191 and E192 (RxKE motif) make base-specific contacts with DNA. R189 makes a bidentate hydrogen bond to the inner G base (Figure 3A and B). Nζ atom of K191 makes hydrogen bonds to O6 and N7 atoms of the outer guanine (Figure 3C). The side chain of E192 bridges the N4 atoms of both cytidines (Figure 3B and C). K191-E192 of PfoI could be aligned with the first two residues of the conserved R-(D/E)R motif (Figure 1A). Structural comparison shows that RxKE motif of PfoI spatially coincides with R-(D/E)R motif of NgoMIV (Figure 3B and C) and EcoRII, where R189 corresponds to the second R of R-(D/E)R motif (Supplementary Figure S6A and S6B).

Contacts to GG dinucleotide are made also in the minor groove. Q105 makes H-bonds to N2 atoms of both G bases using side chain and main chain carbonyl oxygen atoms (Figure 3B and C). Comparison of the crystal structures of DNA-bound CCGG-family REs reveals that a similar contact involving either an asparagine or glutamine (N/Q) side chain is also present in Ecl18kI (14), EcoRII (13), PspGI (12), SgrAI (9), BsaWI (10) and AgeI (11) (Figure 1A).

### Outer TA bp recognition

In the major groove recognition of the outer T:A base pair in PfoI–DNA complex is achieved by the amino acids from the C-terminal DNA-recognition loop 237–250 (Figure 3D). Q247 residue makes bidentate hydrogen bonds to N6 and N7 atoms of adenine (5′-T|CCGGA). The methyl group of the complementary T base (5′-T|CCGGA) makes van der Waals contact with Nη2 atom of R257 residue. Additionally, K191, which is also involved in the recognition of the outer G, is engaged in a water-mediated hydrogen bond to the O4 atom of T base.

In the minor groove Nζ atom of K109 residue makes a hydrogen bond to O2 of outer T base (Figure 3D). This side chain is located on the same N-terminal helix α6, that carries catalytic residue E113 and Q105 that interacts with the CCGG-tetranucleotide (Figure 3B and C). CCGG-REs that interact with 6 bp target sequences make hydrogen bonds to the outer base pair in the minor groove. Interestingly, the position of this interacting residue in the sequence is conserved, also in the case of PfoI K109 (Figure 1A).

### The central base pair is flipped out of DNA duplex

**Figure 4.**
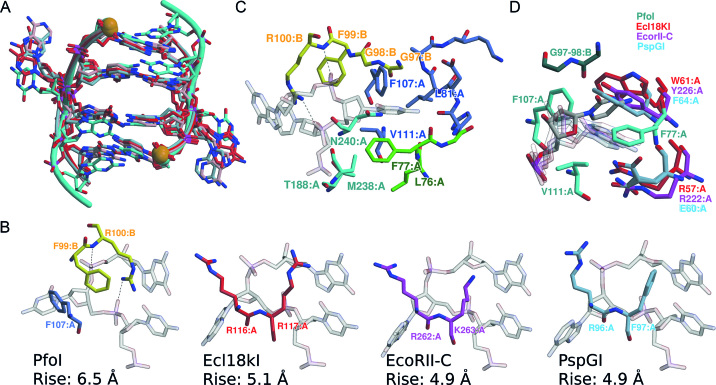
Central base binding pocket of PfoI. (**A**) Superimposed CC(N)GG tetranucleotides from the crystal structures of PfoI, Ecl18kI (PDB ID 2FQZ), EcoRII (PDB ID 3HQG) and PspGI (PDB ID 3BM3). DNA from PfoI–DNA complex is colored cyan, other DNAs are colored as in (B). Scissile phosphates are shown as orange spheres. (**B**) The side chains displacing the flipped base in PfoI, EcoRII, Ecl18kI and PspGI. The side chains of both PfoI monomers are colored blue and yellow (as in Figure 1B and C). Hydrogen bonds between R100 and phosphate backbone of DNA are shown as dashed lines. The Rise distances (calculated using the w3dna web server (39)) of the C:G and G:C inner bp steps of the corresponding DNAs are shown. (**C**) Interaction of PfoI with the central flipped base. PfoI structural elements are colored like in Figure 1B (residues 78–111 - blue and yellow from different protein monomers, N-subdomain - green and C-subdomain residues - cyan). (**D**) Comparison of the flipped base pair pocket of PfoI (dark cyan and cyan for different protein monomers, flipped C7 base is gray) with Ecl18kI (red), EcoRII-C (magenta) and PspGI (light blue).

In the PfoI–DNA complex the central nucleotides of the recognition sequence are extruded from DNA stack (Figure 1C). DNA bound in PfoI complex is essentially B-form DNA, except for the strongly distorted central flipped base pair and base pairs at the G-G step (5′-T|CCNGGA) (Figure 4A, Supplementary Figure S7). The inner G:C pairs exhibit a striking propeller twist, and the outer G:C pairs are strongly buckled (Supplementary Figure S7A). DNA bound by PfoI is bent and the distance between the scissile phosphates in the PfoI-bound DNA becomes 18.1 Å, which is close to 18.0 Å that was measured in the DNA oligoduplex bound to Bse634I (PDB ID 3V20 (18)). This distance is slightly larger than for B-form CCGG-tetranucleotide containing DNA, or for the oligoduplexes observed in the crystal structures of Ecl18kI, EcoRII-C and PspGI (12–14). While DNA conformations near the scissile phosphate atoms are similar in the complexes with PfoI and other nucleotide flipping REs, conformations differ drastically for the inner C:G pairs. The values of local base-pair step parameters for C:G and G:C bp step in complexes with flipped DNA (CC(N/W)GG) differ significantly between PfoI and other REs (Supplementary Figure S7). The largest difference is observed in Rise value, which is ∼6.5 Å in PfoI, compared to 4.9–5.1 Å for the other REs (Figure 4B and Supplementary Figure S7C).

The flipped nucleotide is positioned in the binding pocket and amino acid residues from both subunits contribute to its binding. Residues 76–81, 107, 111 from the N-subdomain and residues 188 and 238–240 from the C-subdomain of one subunit participate in the binding of the flipped nucleotide along with the loop 96–100 from the other subunit (Figure 4C). Residues 78–111 are swapped between two monomers of PfoI–DNA complex, whereas in the apo-PfoI structure this structural element is bound to the same subunit (Figure 1B and C, Supplementary Figure S4B).

The loop 78–111 forms a clamp that pushes the flipped base and fixes it in the pocket formed mainly by the other protein chain (Figure 4C). The other structural element of PfoI, involved in the binding of the flipped base, is the DNA-recognition loop 237–250, that is mostly disordered in the apo-PfoI. In the complex with DNA, residues 238 and 240 form one of the sides of the flipped base pocket (Figure 4C). The binding pocket is rather spacious in PfoI, as it should adopt not only 2′-deoxycytidine, as in the crystal structure, but also both purine 2′-deoxynucleosides.

In the PfoI–DNA complex the side chains of F99-R100 displace the flipped base from the minor groove (Figure 4B). The importance of these residues was tested by replacement with alanine. Analysis of the F99A and R100A mutants and of the double mutant F99A-R100A demonstrated that arginine side chain replacement renders the protein inactive (Table 2). Replacement of the F99 prevents the complete cleavage of λ DNA (Table 2). Mutations reduced the DNA binding of PfoI (Supplementary Figure S5C). Complexes formed by mutant proteins with the specific oligoduplex have reduced mobility in the gel, which is similar to the mobility of the complexes formed with the unspecific oligoduplex (Supplementary Figure S5D) suggesting that residues F99 and R100 are important for the formation of a compact/closed specific complex.

The pocket for the flipped base differs from that of Ecl18kI, EcoRII and PspGI (Figure 4C and D). In PfoI the position of the flipped base is locked away from the base pair stack of DNA by the residues F107 and F99-R100 coming from different subunits (Figure 4B). Residues with possibly similar function R116-R117 of Ecl18kI, R96-F97 of PspGI and R262-K263 of EcoRII occupy different positions in space, and only backbone atoms of F107 coincide with the invariant arginine residue of Ecl18kI, PspGI and EcoRII (Figures 1A and 4B).

### Plasmid DNA cleavage

CCGG-family members differ in target site requirements (11). NgoMIV (8), Cfr10I (57), Bse634I (7,58,59), BsaWI (10), Ecl18kI (60) and SgrAI (61) form stable or transient tetramers and require an extra copy of the target site for optimal cleavage. In order to determine whether PfoI requires an additional target sequence for optimal cleavage, we analyzed PfoI cleavage of a single-site plasmid in the presence of a specific oligoduplex. Under multiple turnover conditions (E<S), linear DNA, the final reaction product, accumulates in parallel with the reaction intermediate nicked plasmid DNA, indicating that PfoI can cleave both DNA strands in a single binding event (Supplementary Figure S8A). Under saturated conditions (E>S), the final product of 1-site DNA plasmid cleavage is a linear FLL form with both DNA strands cut at the PfoI recognition site (Supplementary Figure S8B). Addition of the specific oligoduplex decreases rather than stimulates plasmid cleavage (Supplementary Figure S8C). Therefore, we conclude that PfoI like other orthodox Type IIP enzymes cleaves DNA as a dimer.

## DISCUSSION

### Permutations in PfoI active site

CCGG-family enzymes use conserved DNA recognition elements and active sites to recognize and cut both intact CCGG and interrupted CCNGG sequences. The typical PD…D/ExK catalytic motif identified in many type II REs is permuted in the CCGG-family into the PD…K…D/E variant by an interchange of the positions of the lysine and acidic D/E residue. This active site permutation, first identified in Cfr10I (6), was later established in other CCGG-family REs (16,17). The glutamate residue (underlined) in the PD…K…D/E motif is conserved in most CCGG-REs, but it can be replaced by a glutamine (Kpn2I), or aspartate (BsaWI and AgeI) (Figure 1A). The crystal structure of PfoI revealed E^113^…E^167^xD^169^…K^187^ catalytic motif. This order of active site residues results from yet another permutation of the canonical active site motif. The positions of aspartate D169 and lysine K187 are conserved in the protein sequences of other CCGG-family REs. In contrast, the positions of E113 and E167 are shifted in the sequence (Figures 1A and 2A).

### Role of active site residues

The active site of PfoI contains one Ca^2+^ ion, similarly to NgoMIV-substrate DNA complex (PDB ID 4ABT, Figure 2). Binding of only a single metal ion in the active site is not uncommon, particularly when Ca^2+^ substitutes for Mg^2+^ (54). However, in the co-crystal structures of the CCGG family REs SgrAI ((62), PDB ID 3MQY) and NgoMIV ((8), PDB ID 1FIU) that were obtained in the presence of Mg^2+^, two Mg^2+^ ions are present in each active site (Supplementary Figure S9). Positions of Mg^2+^ ions in NgoMIV and SgrAI crystal structures coincide with metal sites A and B in the crystal structures of REs of PD…D/ExK family (56) implying that PfoI can also bind two Mg^2+^ ions.

The position of the metal B is occupied by a water molecule in the PfoI-DNA co-crystal structure (Figure 2A and C). This water molecule interacts with the leaving group oxygen of the scissile phosphate, the side chain carboxylate atoms of residues E167 and D169, and another water molecule. As Mg^2+^ ions have a preference for octahedral coordination, it is possible that Mg^2+^ ion in metal B position may recruit an additional water molecule that could be possibly anchored by residue E113. This residue would thus become an indirect metal ion ligand, since the distance 5.4 Å between E113 and metal site B allows a water-mediated interaction of E113 and metal ion B (Figure 2A, cyan). Similarly, in NgoMIV only D169 directly coordinates both metal ions while other two carboxylate residues E70 and E201 interact with metal ions via water molecules (Supplementary Figure S9). Small changes in the relative positions of the metal coordinating residues E70 and E201 are observed when comparing Ca^2+^-bound and Mg^2+^-bound NgoMIV–DNA complexes (Supplementary Figure S9C). Therefore we assume that PfoI active site residues could also bind up to two Mg^2+^ ions per protomer.

Structural and mutagenesis data are consistent with the canonical roles for the active site residues, despite their permutation in the amino acid sequence. However, the catalytic role of E113 is not immediately clear from the structure. Experimentally, the E113A substitution drastically reduces DNA binding and cleavage (∼50-fold) (Table 2). For the wild-type enzyme, the glutamate carboxylate group (Oϵ2 atom) is ∼7.3 Å from metal A site and ∼5.4 Å from the proposed metal B site, indicating that there is no direct contact with the metal ions (Figure 2A). However, E113 could still act as an indirect ligand of the predicted metal B, which should enhance catalysis and promote leaving group departure (Figure 2A). Alternatively, E113 could be required to keep K109 positioned for semi-specific minor grove contacts with outer bp of the recognition sequence, and indeed the E113-K109 interaction has a direct counterpart in AgeI (E94-R90) and SgrAI (D100-K96) (Figures 1A and 2C). The two roles are not mutually exclusive. If both are relevant, the E113-K109 pair could be involved in coupling DNA recognition and catalysis.

Another pair of residues with possible role in coupling of catalysis and sequence readout is the catalytic lysine residue (replaced by alanine in the PfoI co-crystal structure with DNA), which interacts through the water molecule with the last R residue of the CCGG recognition R-(D/E)R motif. This interaction is observed in complex structures of NgoMIV (PDB ID 1FIU) and AgeI (PDB ID 5DWB). In Bse634I-DNA structure only few solvent molecules were modeled due to moderate resolution, however in this structure the distance between catalytic K198 and the corresponding R205 is 4.5 Å, which is in line with the water-mediated hydrogen bond (PDB ID 3V21). Hence, we assume that similar interaction between K187 and R189 in PfoI is also involved in coupling of recognition and catalysis.

### CCGG recognition

The conserved amino acid residues R-(D/E)R involved in the recognition of the CCGG sequence are located at the N-terminus of the conserved α helix (17,63). Variations of this motif are observed within the CCGG-family (Figure 1A). The CCGG recognition motif of EcoRII contains K in the position of the first R (K328-D329-R330). Yet another permutation of the R-(D/E)R motif, E173-R174-K200 is observed in AgeI. In that case the role of the first arginine is played by the lysine K200 side chain coming from an additional structural element (11). PfoI amino acid residues R189-x-K191-E192 interacting with the CCGG sequence are located in the same region, but differ from the consensus R-(D/E)R residues in the position of the second R (Figure 1A). Due to the different position in the sequence, the approach of the R189 side chain to the Hoogsteen edge in PfoI is different compared to other family members. In the canonical situation, the guanidino Nϵ and Nζ1 atoms interact with the guanine O6 and N7 atoms, respectively. In the alternatively anchored R189 of PfoI, the role of the nitrogen atom hydrogen bond donors is reversed with respect to NgoMIV and EcoRII-C (Figure 3B and Supplementary Figure S6A and S6B). Permutations of the active site and CCGG recognition motifs of PfoI demonstrate the importance of structural rather than sequence conservation of key active site and DNA recognition residues.

### PfoI outer bp recognition shares similarities with AgeI

Two PfoI structural elements, DNA recognition loop (residues 237–259) and CCGG-recognition motif, contribute to the recognition of the outer T4:A10 base pair from the major groove (Figure 3D). Residues Q247 and R257 of the DNA recognition loop make bidentate hydrogen bond to A base and van der Waals contact with methyl group of T base, respectively. Additionally, T base is recognized through a water mediated hydrogen bond from K191 of CCGG-recognition motif. Similar DNA recognition loop (residues 197–224) is present in AgeI that is specific for the A|CCGGT sequence and it contributes to the recognition of the outer A:T base pair (Supplementary Figure S6C) (11). K200 residue of this loop is also involved in CCGG recognition, similarly to a double role of K191 of PfoI. Different structural elements contribute to the recognition of outer G:C bp by NgoMIV specific for the 5′-G|CCGGC sequence (8). In the case of Bse634I (5′-R|CCGGY) the main role in the recognition of the outer base pair is played by the indirect readout (18).

An undiscriminating contact of outer base pair in the minor groove similar to K109 in PfoI is also present in other CCGG-REs that interact with 6 bp recognition sequence (Figure 1A) (10). These residues make hydrogen bond to pyrimidine atom O2 in the minor groove. Interestingly, asparagine and glutamine in Bse634I and NgoMIV, respectively, are directed toward the other DNA strand as K109 in PfoI, because C or T bases are located on the other strand in recognition sequences of these REs (8,18). In contrast to K109 in PfoI, K96 in SgrAI does not form a hydrogen bond with O2 atom (9).

### New way to mimic CCGG tetranucleotide

Structurally characterized Ecl18kI, EcoRII, PspGI that recognize interrupted CCNGG or CCWGG sequences, flip the central nucleotide and restore the base stacking interactions between the adjacent CC:GG blocks to mimic uninterrupted CCGG sequence (12–14). Such change in DNA conformation allows these enzymes to use conserved recognition elements and active sites for interaction with CCNGG and CCGG sequences (12–14,20). Surprisingly, DNA conformation in PfoI complex is different (Figure 4A, Supplementary Figure S7). While the central nucleotide is also flipped, DNA is not compressed, like in EcoRII-C, PspGI and Ecl18kI, but the DNA strand carrying GG step is bent toward the major groove starting from the flipped base. Therefore, the inner G:C pairs do not stack against each other and exhibit unusually large distortions that differ from the DNA conformation in other base-flipping REs (Supplementary Figure S7). As a result, the scissile phosphodiester bonds in PfoI are positioned at a distance comparable to that in the CCGG sequence. This finding shows that different DNA conformational changes can lead to the CCGG sequence mimicry. It cannot be excluded that the different base pair conformations in the PfoI complex and the Ecl18kI, PspGI and EcoRII complexes may be related to the variation of the CCGG recognition motif in these REs (conserved between Ecl18kI and PspGI, altered in EcoRII and drastically altered in PfoI).

### Different binding of the flipped nucleotide

In the complexes of nucleotide flipping CCGG-REs with DNA, the flipped bases are always accommodated in dedicated binding pockets (Figure 4C and D). In Ecl18kI, EcoRII and PspGI nucleotide binding pockets are similar and are formed by two N-terminal helices (12–14). The PfoI nucleotide binding pocket is different; it is formed by the residues coming from both protein subunits and involves not only N-subdomains but also the DNA recognition loop of the C-subdomain (Figure 4C).

In PfoI, the flipped bases are wedged in between the almost perpendicular phenyl rings of F77 and F107, in herringbone arrangement (64) (Figure 4C). In contrast, the flipped bases are oriented parallel to the aromatic rings of W61 in Ecl18kI, Y226 in EcoRII and F64 in PspGI (Figure 4D). Fluorescence experiments of PfoI–DNA complexes using 2AP in the central position of the recognition sequence indicated that the relative increase in intensity is much larger for PfoI (∼1000-fold) than for other CCGG-family base flipping REs (6.5- to 64-fold) (20,21). Differences in quenching may be explained by the different orientations of the aromatic rings in the nucleotide binding pockets resulting in the more drastic response of the conformational probes to flipping by PfoI (almost perpendicular orientation, no quenching, Figure 4C) compared to Ecl18kI, EcoRII or PspGI (almost parallel orientation, quenching, Figure 4D).

### A proposed model for the pathway leading toward the specific complex

**Figure 5.**
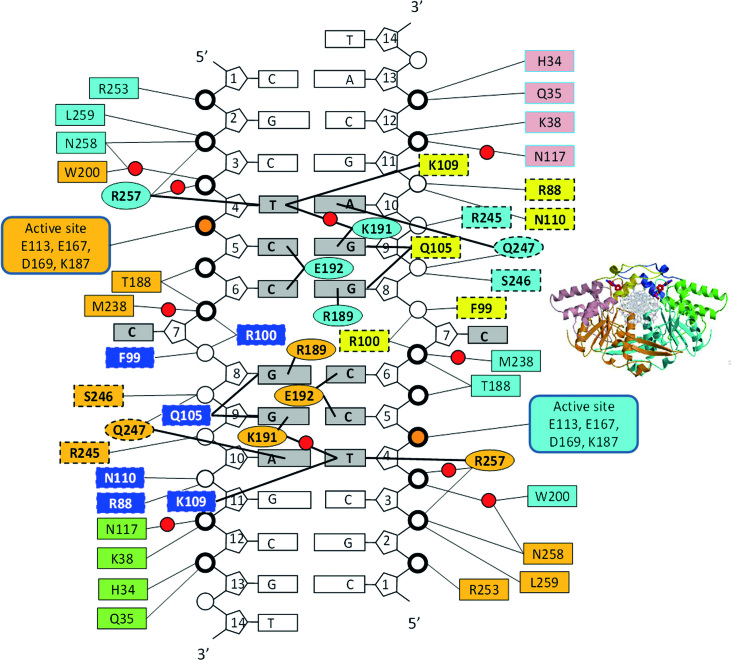
Schematic representation of PfoI-DNA contacts. Amino acid residues making contacts to backbone phosphates are shown in boxes and are colored as in Figure 1B. Residues that change their positions upon DNA binding are shown within dashed boxes. Residues making contacts in the major groove are shown in ellipses. Backbone phosphates, which make contacts with the residues that do not change their positions in apo- or DNA-bound PfoI, are marked by a thick line. Scissile phosphates are colored orange. Red spheres represent water molecules.

Analysis of the DNA backbone contacts in the specific PfoI–DNA complex reveals that PfoI makes many contacts to DNA backbone within and outside target sequence (Figure 5). This could help, probably, to explain the absence of DNA in crystals of PfoI with shorter oligoduplexes SP11 and SP12. Non-specific contacts with DNA backbone found in crystal structure up to 3 bp outside of the recognition sequence. Therefore, 11 and 12 bp oligoduplexes were too short to form a stable specific complex with PfoI, but may have promoted its crystallization.

Many of the backbone contacts, namely all contacts outside the recognition sequence and with 5′-TCC part of the recognition site, are made by the PfoI elements that do not undergo large conformational changes upon DNA binding (Figure 5). The remaining contacts within the recognition sequence are made by the elements that change their conformation, namely helix α6 and DNA recognition loop 239–249 (Figure 5). We assume that the first part of the backbone contacts may be present also in the non-specific complex. After binding to the specific sequence and making base specific contacts in the major groove, the minor groove contacts may be formed, including the interactions of Q105 with the inner G-bases (5′-T|CCGGA, Figure 3B and C). Insertion of helix α6′ into the minor groove affects protein and DNA conformation. In the protein, the conformation of the hinge at the residue G112 changes, so that helices α6′ and α6 (residues 99–104 and 110–127, Supplementary Figure S1A) combine into long helix α6 (residues 101–127, Supplementary Figure S1B) that is homologous to the N-terminal helices in Ecl18kI, PspGI and EcoRII. In the DNA, the helix insertion widens the minor groove, and allows R100 and F99 to push the central base out of the duplex where R100 makes a stacking interaction with the inner G (5′-T|CCNGGA, Figure 4B). Upon DNA binding, the previously disordered DNA recognition loop 239–249 gets ordered, and may help to fix the flipped base in the pocket.

### PfoI is the most diverged member of the CCGG family

REs of the CCGG-family recognize a set of 4–8 bp target sequences that contain a common CCGG/CCNGG core. PfoI recognizes the 7 bp sequence 5′-T|CCNGGA and shares the cleavage position with other CCGG-family members. However, PfoI protein sequence shows no significant similarities to ones of other family members. Crystal structures of PfoI presented here revealed structural similarities with CCGG-family members. However, PfoI shows a permutation of catalytic and CCGG recognition residues in comparison to other family members as well as different DNA conformation. In summary, the PfoI structure demonstrates the diversity and versatility of structural mechanisms for the recognition of related DNA sequences.

## DATA AVAILABILITY

Atomic coordinates and structure factors for the reported crystal structures have been deposited with the Protein Data bank under accession number 6EK1 (apo-PfoI) and 6EKO (PfoI–DNA complex).

## Supplementary Material

Supplementary Data
