# Supplementary material for: Unique mechanism of target recognition by PfoI restriction endonuclease of the CCGG-family
Source: Nucleic Acids Res. 2018 Nov 16;47(2):997–1010. doi: 10.1093/nar/gky1137 (PMC6344858; doi:10.1093/nar/gky1137)
Supplement: Supplementary Data [file gky1137_supplemental_files.pdf]

Supplemental Figures and Tables accompanying manuscript

Unique mechanism of target recognition by PfoI restriction endonuclease of the CCGG-family

Giedre Tamulaitiene[1, †,*], Elena Manakova[1, †], Virginija Jovaisaite[1], Gintautas Tamulaitis[1], Saulius Grazulis[1], Matthias Bochtler[2,3] and Virginijus Siksnys[1,*]

[1] Institute of Biotechnology, Vilnius University, Sauletekio al. 7, LT-10257, Vilnius, Lithuania

[2] Dept. of Structural Biology, International Institute of Molecular and Cell Biology, Trojdena 4, 02-109 Warsaw, Poland

[3] Dept. of Bioinformatics, Institute of Biochemistry and Biophysics, Polish Academy of Sciences, Pawinskiego 5a, 02-106 Warsaw, Poland

* To whom correspondence should be addressed. Tel: +370-5-2234357; Fax: +370-5-2234367; Email: eigie@ibt.lt

Correspondence may also be addressed to Virginijus Siksnys. Tel. +370-5-2234359; Fax: +370-5-2234367; Email: siksnys@ibt.lt

[†] The authors wish it to be known that, in their opinion, the first two authors should be regarded as joint First Authors.

Present Address: [Virginija Jovaisaite], Novartis Institutes for BioMedical Research, CH-4002, Basel, Switzerland.

Supplementary Table S1. DNA oligoduplexes used in this study.

Name	Sequence*	Comment
SP14	5'-CGCTCC**C**GGAGCGT-3'<br>3'-TGCGAGG**C**CCTCGC-5'	Crystallization of PfoI K187A
SP12	5′–GCTCC**C**GGAGCT–3′<br>3′–TCGAGG**C**CCTCG–5′	Crystallization of apo PfoI
SP11	5'-GCTCCGGGAGC-3'<br>3'-CGAGGCCCTCG-5'	Crystallization of apo PfoI
SP25	5'-CGCACGACTTCCTGGAAGAGCACGC-3'<br>3'-GCGTGCTGAAGGACCTTCTCGTGCGTTG-5'	Cognate, PfoI
NSP25	5'-CGCACGACTTGTCACAAGAGCACGC-3'<br>3'-GCGTGCTGAACAGTGTTCTCGTGCGTTG-5'	Non-cognate, PfoI
SP23	5'-CGCACGCCTTCCTGGAAGCACACTA-3'<br>3'-GCGTGCGGAAGGACCTTCGTGTGAT-5'	In-trans stimulation of plasmid cleavage
SP14-GF	5'-TAGCTCCAGGACGA-3'<br>3'-TCGAGGTCCTGCTT-5'	Gel filtration of PfoI
SP17	5'-TTCGCTCC**C**GGAGCGA-3'<br>3'-AGCGAGG**C**CCTCGCTT-5'	Oligoduplex used for DLS

*The PfoI recognition sequence (5'-T|CCNGGA) is underlined. Unpaired bases of the PfoI recognition sequence are in bold face.

Supplementary Table S2. Estimation of PfoI oligomeric state by gel filtration.

Sample	MW* in low salt conditions, kDa	MW in high salt conditions, kDa
PfoI + SP14-GF	50.2	48.6
PfoI	46.9	49.3

*Monomer MW is 35.1 kDa.

Supplementary Table S3. Estimation of PfoI oligomeric state by DLS.

Sample	MW* estimation, kDa	Particle diameter, nm
PfoI + SP14	58±10.4 (PDI** 15.8%)<br>58.0±9.6 (PDI 15%)	6.752±1.127<br>6.752±1.055
PfoI K187A + SP17	64.6±11.0 (PDI 15.9%)<br>64.6±12.1 (PDI 16.7%)	7.071±1.154<br>7.071±1.237

*Monomer MW is 35.1 kDa.
**PDI is the Polydispersity Index

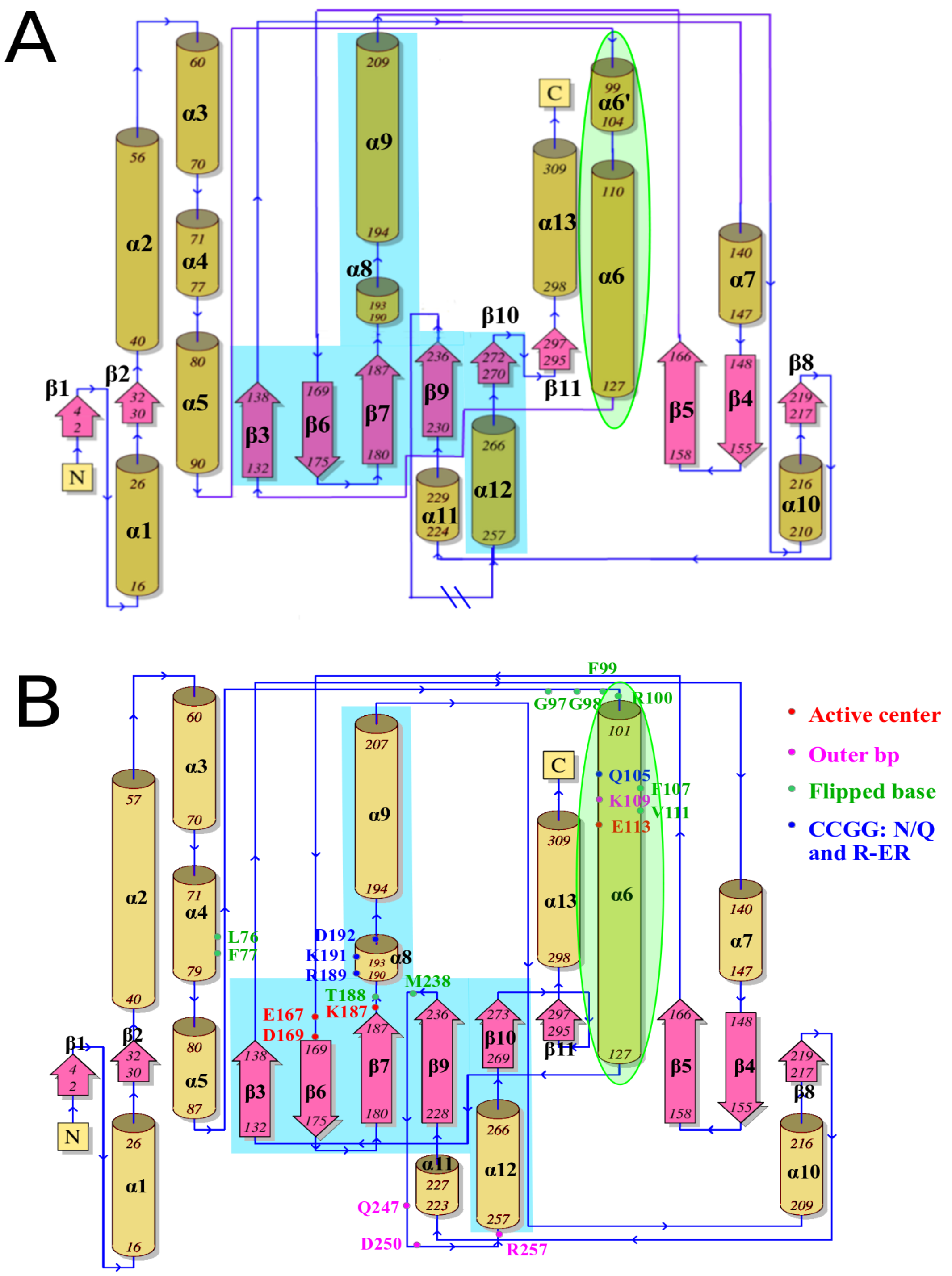

Supplementary Figure S1. Secondary structure of apo (**A**) and DNA-bound (**B**) PfoI. The conservative structural core representing RE fold is highlighted cyan. The α6 helix that is broken in the apo-PfoI and becomes longer in PfoI-DNA complex is highlighted green. Residues that form active center and participate in DNA recognition are marked on the corresponding secondary structure elements in B.

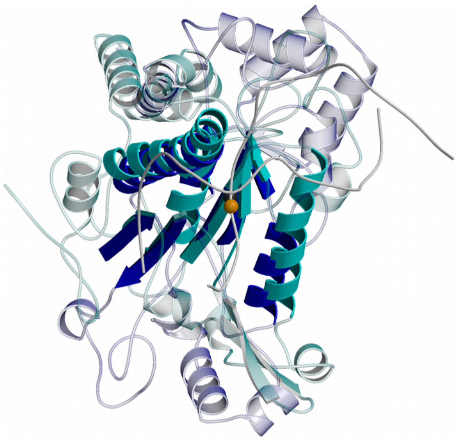

Supplementary Figure S2. RE fold core elements of NgoMIV (colored blue, PDB ID: 4ABT) superimposed on PfoI complexed with DNA (colored cyan). DNA oligoduplex is shown as grey trace, and the scissile phosphate is shown as an orange sphere.

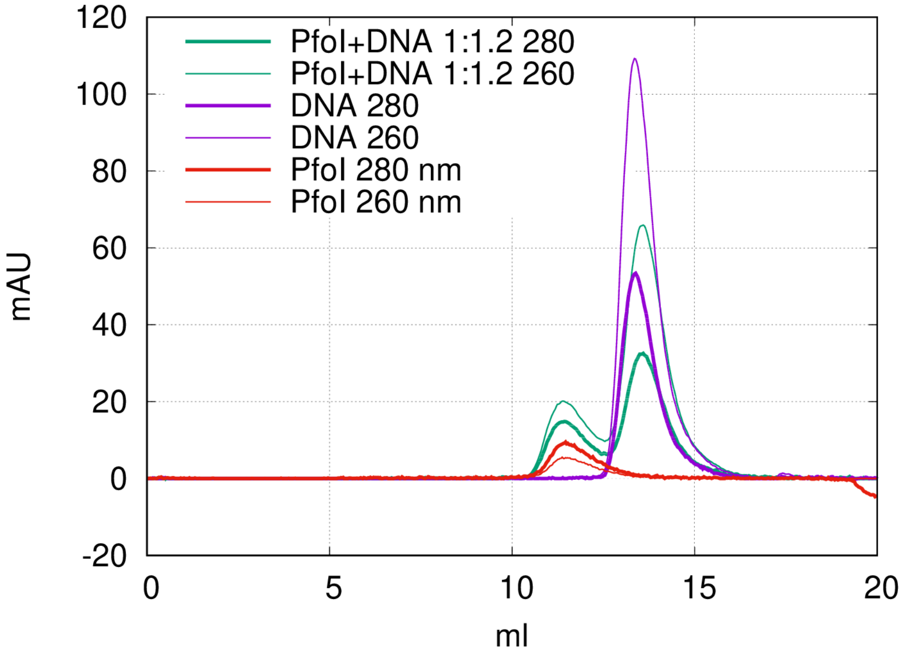

Supplementary Figure S3. Elution profiles of PfoI complex with SP14-GF (Supplementary Table S1) oligoduplex (green curves) and apo PfoI (red curves). Gel filtration experiments are performed as described in "Materials and Methods". Thin curves correspond to the absorption at 260 nm, whereas thick curves are absorption at 280 nm. Apo PfoI gel filtration was performed in buffer containing 300 mM of salt and overlaid with the elution profile of PfoI-DNA complex in lower salt (150 mM) buffer.

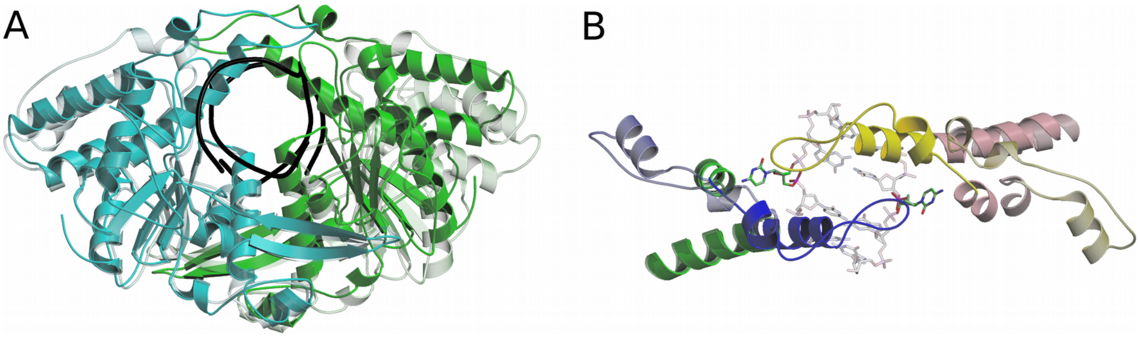

Supplementary Figure S4. Conformational changes of PfoI induced by binding of specific DNA oligoduplex. **A**, Dimer of apo-PfoI (transparent) superimposed on PfoI-DNA complex (monomers colored cyan and green) by residues 113-230 of a monomer colored cyan. DNA is shown as black trace. **B**, Rearrangement of N-domains upon DNA binding. Residue range 72-126 of both subunits of PfoI-DNA complex as well as of apo-PfoI are colored according to the scheme in Figure 1B and 1C. Apo-PfoI residues are transparent. DNA is shown as ball-and-stick and transparent, flipped C7 bases are colored green.

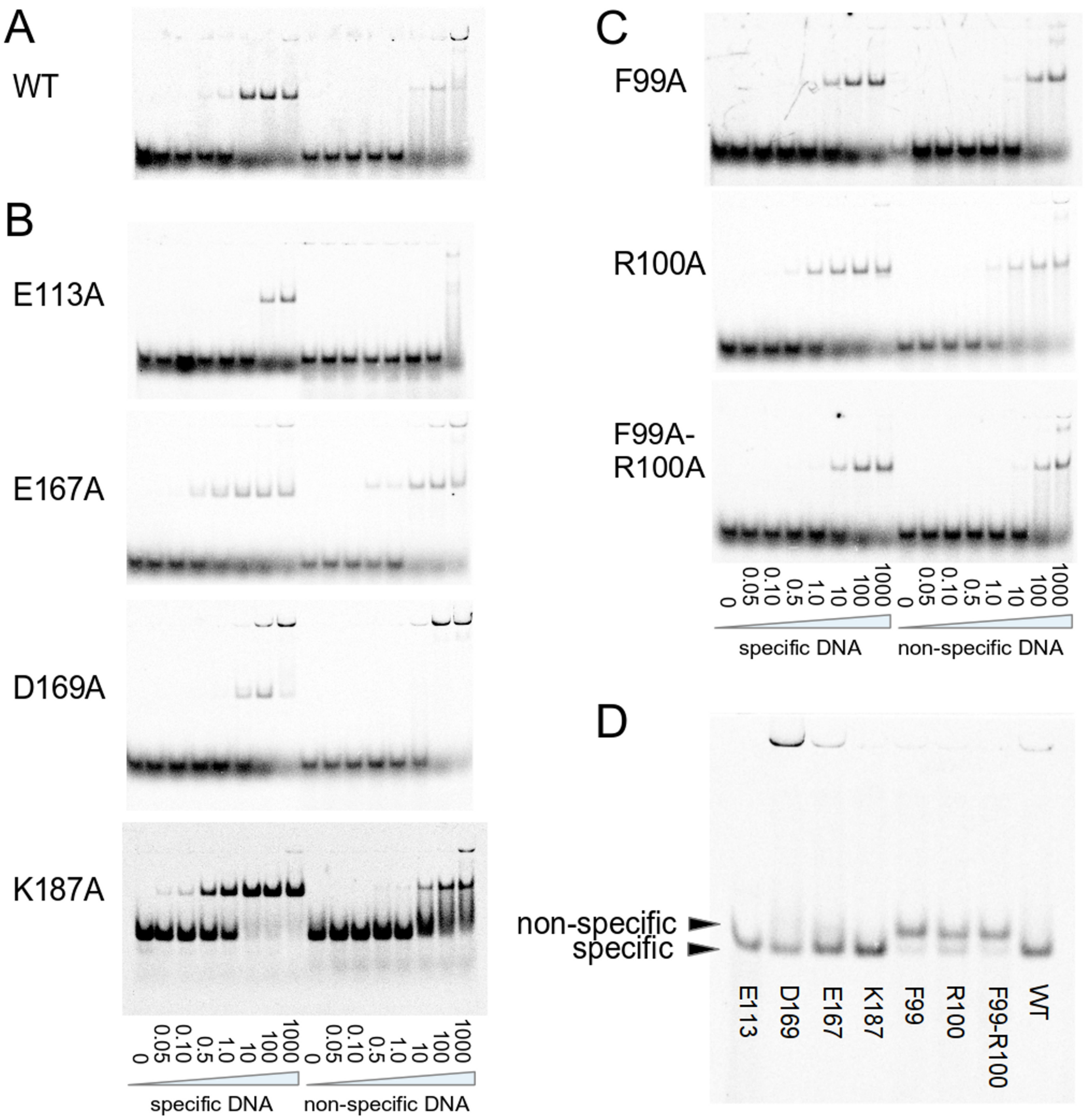

Supplementary Figure S5. Specific and unspecific oligoduplexes binding by wt PfoI and PfoI mutants. The samples were separated by native PAGE and analyzed as described in 'Materials and Methods'. Concentrations of PfoI dimers (nM) are shown below gels. Specific and non-specific oligoduplexes used in experiments were of the same length (Supplementary Table S1, SP25 and NSP25, correspondingly). DNA concentration was 0.2 nM. **A**, WT PfoI. **B**, Active center mutants. **C**, Mutants of the flipped base pocket. **D**, Comparison of the mobility of complexes formed by PfoI mutants with specific DNA SP25 (Supplementary Table S1). Protein concentration was 100 nM (dimer).

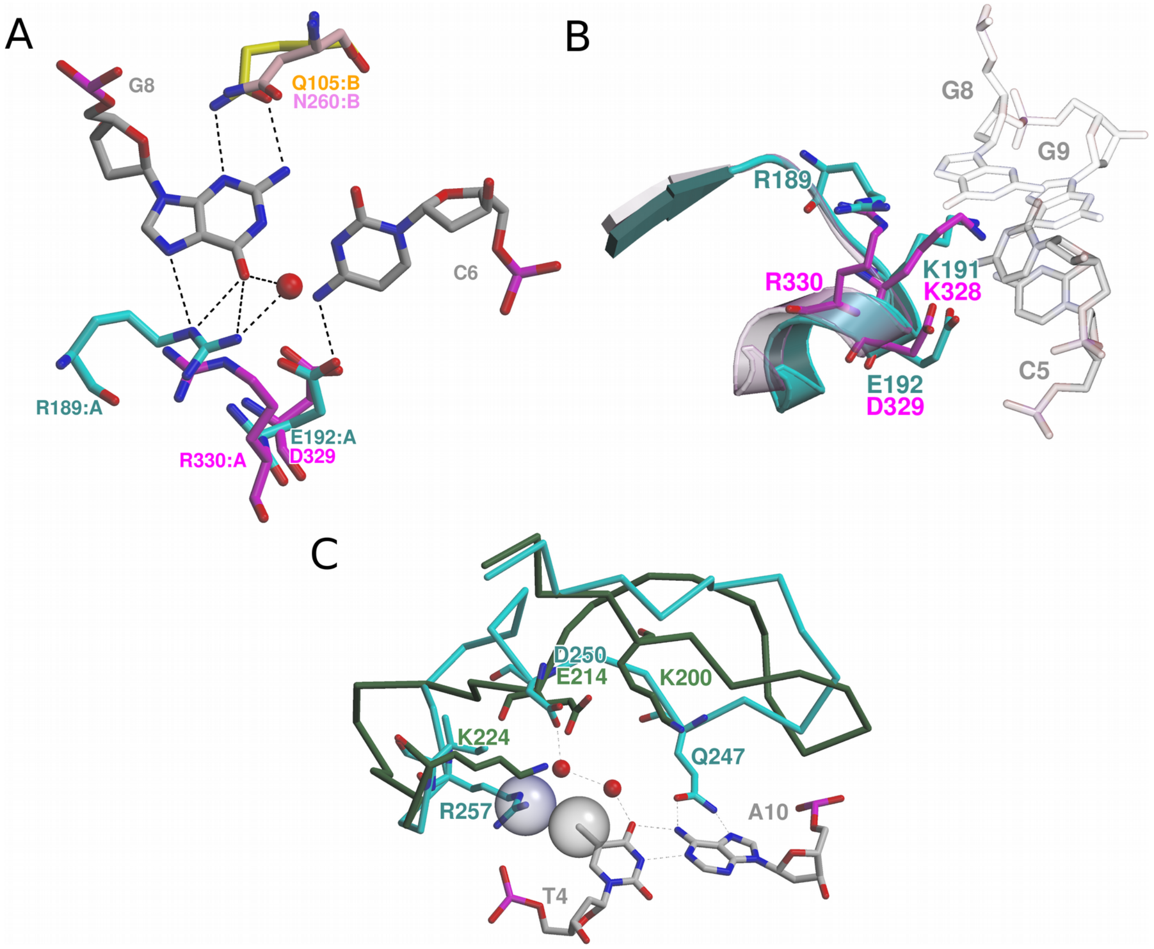

Supplementary Figure S6. Specific DNA recognition by PfoI compared with EcoRII-C (A and B) and AgeI (C). Water molecules are shown as red spheres. H-bonds are marked by dashed lines. **A**, Recognition of the inner C:G base pair by PfoI (residues from both subunits are colored cyan and yellow, as in Figure 1B and 1C) and EcoRII-C (EcoRII side chains from different subunits are colored pink and magenta). Base pair is shown in grey. **B**, Superimposed CCGG-tetranucleotide recognition motive residues of PfoI (cyan) and EcoRII-C (magenta). Side chains of the residues specifically interacting with CCGG-tetranucleotide are shown in ball-and-stick representation. CCGG base pairs are transparent. **C**, Superposition of the structural elements of AgeI (green) and PfoI (cyan) recognizing outer base pair. Contacting methyl group of T4 and guanidino group of R257 of PfoI are shown in CPK representation.

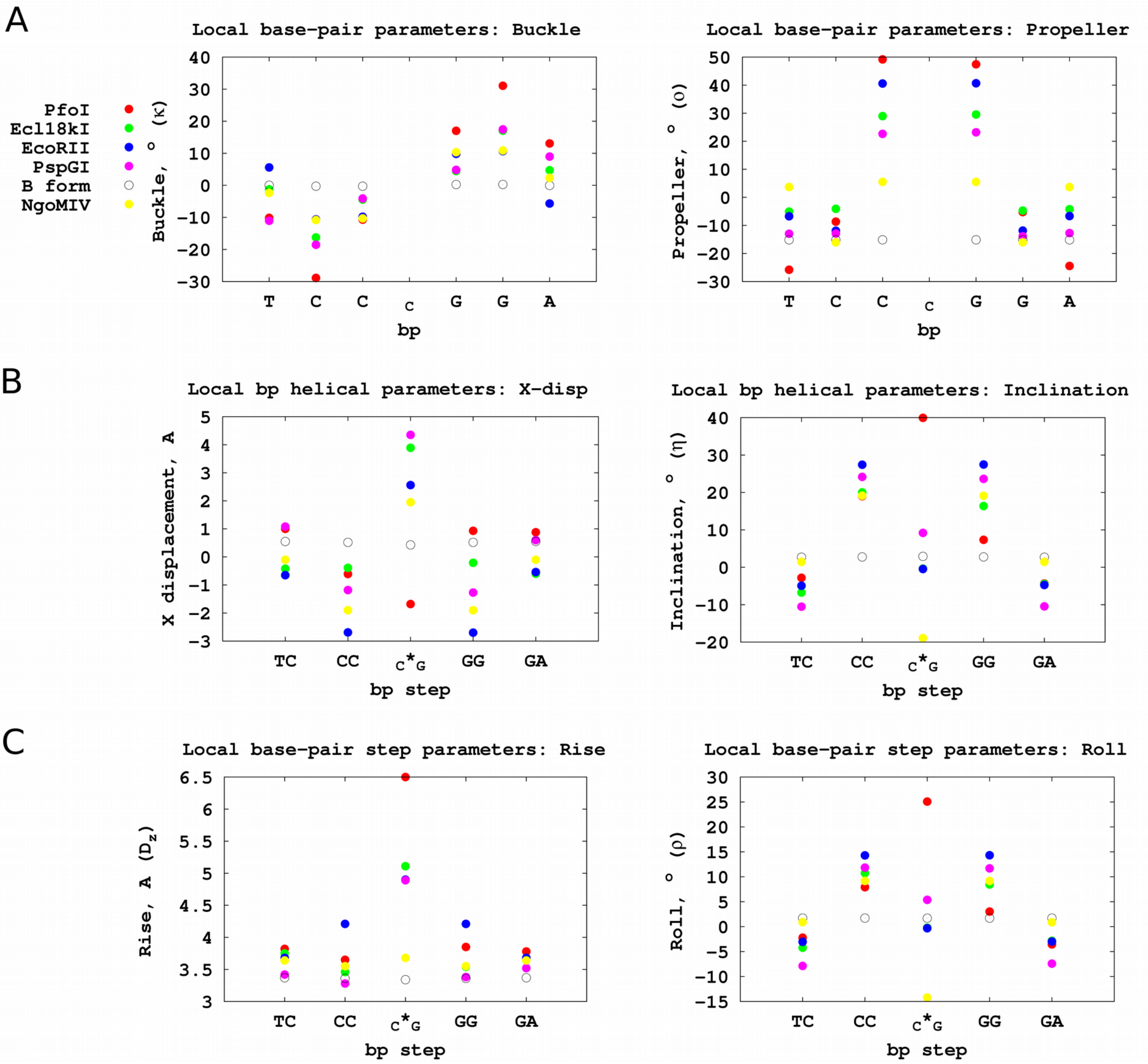

Supplementary Figure S7. Structural parameters of DNA bound by central base flipping REases (PfoI is shown as red circles, Eclk18I - green, EcoRII-C - blue and PspGI - magenta) compared with NgoMIV (yellow circles) and B-form DNA oligoduplex (open circles). DNA parameters were calculated by w3dna. **A**, Local base pair parameters (Buckle and Propeller -Twist). The local base pair parameters of C7 base (shown in lower case) are absent because the bases are unpaired. **B**, Local base pair helical parameters (X-displacement and Inclination). **C**, Local base pair step parameters (Rise and Roll). In B and C, the central C*G base pair is an artefact, that appeared due to the fact that the software ignores the flipped base. Therefore, C*G step corresponds to the C6-G8 apparent step.

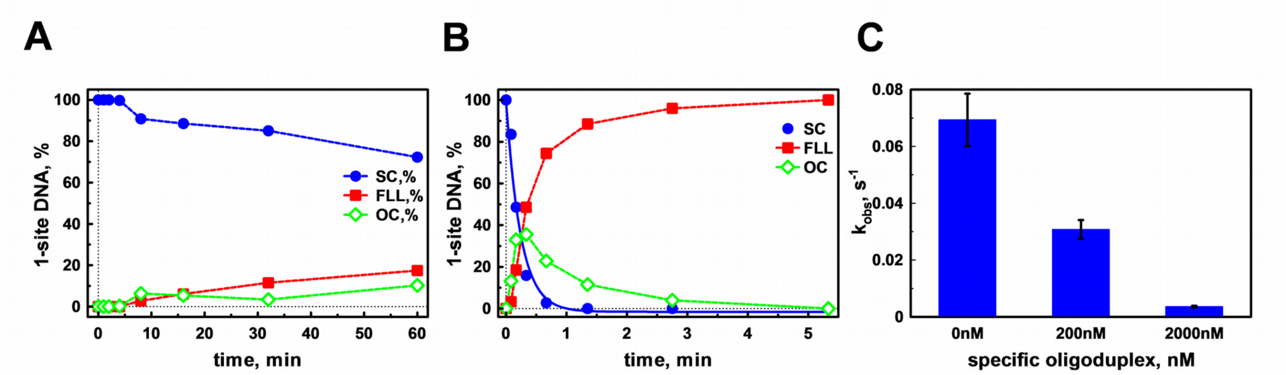

Supplementary Figure S8. One-site plasmid DNA cleavage by PfoI. **A**, Multiple-turnover cleavage of supercoiled pUC18 DNA by *Pfo*I. The reaction contained 0.25 nM wt PfoI and 2.5 nM DNA. **B**, Single-turnover cleavage of supercoiled pUC18 DNA by *Pfo*I. The reaction contained 125 nM wt PfoI and 2.5 nM DNA. **C,** The effect of the *in-trans* specific oligoduplex on the plasmid DNA cleavage by PfoI. The reaction contained 125 nM wt PfoI, 2.5 nM DNA and oligoduplex SP23 (Supplementary Table S1) containing one PfoI target in concentrations 0, 200 and 2000 nM. For (A) and (B) time course of supercoiled (SC), open-circular (OC) and full-length linear (FLL) plasmid DNA measured as described in "Materials and Methods". For (B) and (C) a single exponential was fitted to the time-course of supercoiled (SC) DNA decay to obtain a value for the apparent first-order reaction rate constant k_{obs}.

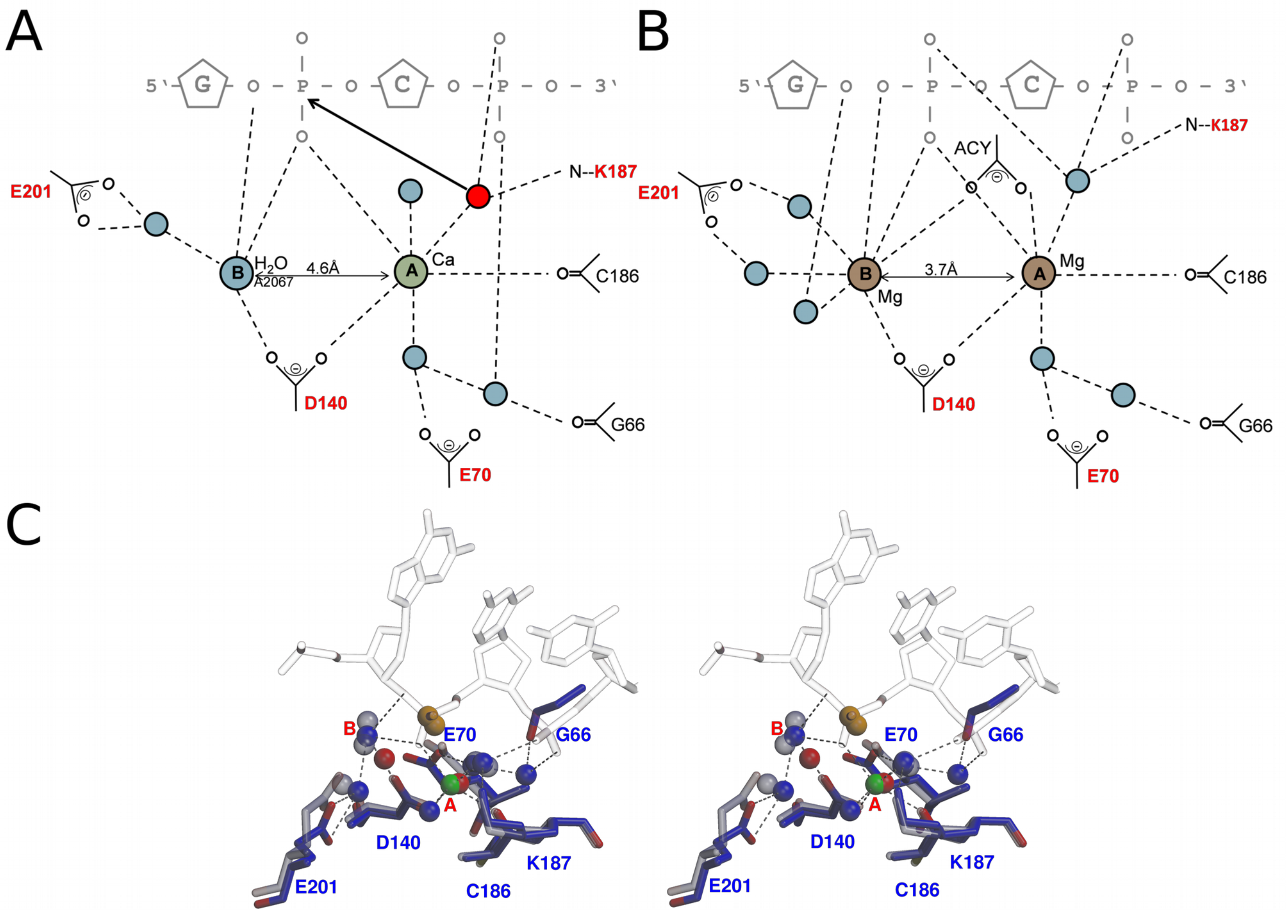

Supplementary Figure S9. Comparison of active center organisation of NgoMIV bound with DNA substrate (PDB ID: 4ABT) and DNA product (PDB ID: 1FIU). Schematic view of the active center of NgoMIV-substrate complex (**A**, PDB ID: 4ABT) and NgoMIV-product complex (**B**, PDB ID: 1FIU). Metal ion sites A and B are designated accordingly, calcium is shown as green circle, magnesium ions - as orange circles, water molecules present in crystal structures are shown as blue circles. The catalytic water molecule is shown red. (**C**) Stereoview of overlaid catalytic residues of NgoMIV-substrate complex (4ABT, blue) with those of NgoMIV-product complex (1FIU, transparent). The scissile phosphate is shown as an orange sphere, Ca^{2+} ion bound in the active center is shown as a green sphere, Mg^{2+} - as red spheres and water molecules are blue spheres.
